# Transcriptome Profiling of the *Phaseolus vulgaris* - *Colletotrichum lindemuthianum* Pathosystem

**DOI:** 10.1371/journal.pone.0165823

**Published:** 2016-11-09

**Authors:** Bilal A. Padder, Kelvin Kamfwa, Halima E. Awale, James D. Kelly

**Affiliations:** Dept. of Plant, Soil and Microbial Sciences, Michigan State Univ., 1066 Bogue St., East Lansing, MI, 48824, United States of America; Università Politecnica delle Marche, ITALY

## Abstract

Bean (*Phaseolus vulgaris*) anthracnose caused by the hemi-biotrophic pathogen *Colletotrichum lindemuthianum* is a major factor limiting production worldwide. Although sources of resistance have been identified and characterized, the early molecular events in the host-pathogen interface have not been investigated. In the current study, we conducted a comprehensive transcriptome analysis using Illumina sequencing of two near isogenic lines (NILs) differing for the presence of the *Co-1* gene on chromosome Pv01 during a time course following infection with race 73 of *C*. *lindemuthianum*. From this, we identified 3,250 significantly differentially expressed genes (DEGs) within and between the NILs over the time course of infection. During the biotrophic phase the majority of DEGs were up regulated in the susceptible NIL, whereas more DEGs were up-regulated in the resistant NIL during the necrotrophic phase. Various defense related genes, such as those encoding PR proteins, peroxidases, lipoxygenases were up regulated in the resistant NIL. Conversely, genes encoding sugar transporters were up-regulated in the susceptible NIL during the later stages of infection. Additionally, numerous transcription factors (TFs) and candidate genes within the vicinity of the *Co-1* locus were differentially expressed, suggesting a global reprogramming of gene expression in and around the *Co-1* locus. Through this analysis, we reduced the previous number of candidate genes reported at the *Co-1* locus from eight to three. These results suggest the dynamic nature of *P*. *vulgaris–C*. *lindemuthianum* interaction at the transcriptomic level and reflect the role of both pathogen and effector triggered immunity on changes in plant gene expression.

## Introduction

Anthracnose caused by *Colletotrichum lindemuthianum* (Sacc. & Magnus)-Briosi & Cavara, is a major pathogen of common bean (*Phaseolus vulgaris* L.) worldwide, but is particularly problematic in temperate regions with cool and humid environmental conditions [1, 2, 3, 4]. The pathogen has been shown to possess high pathogenic variability [5, 6, 7, 8, 9, 10]. Over 100 pathogenic races of *C*. *lindemuthianum* have been reported globally using the 12 differential cultivars and the binary naming system for race identification [11, 12, 13, 14]. As a result of the highly variable nature of the pathogen, there has been frequent break down of genetic resistance in different parts of the world [11, 15, 16, 17, 18, 19, 20].

Seventeen independent loci, *Co-1* to *Co-17*, conditioning resistance have been mapped to eight bean chromosomes (Pv01, Pv02, Pv03, Pv04, Pv07, Pv08, Pv09 and Pv11) [11, 21, 22, 23], and a graphic depiction of the mapped *Co* genes is displayed in Meziadi et al. [24]. Anthracnose resistance is dominant at all loci except the *Co-8* locus, and multiple alleles have been identified at the *Co-1*, *Co-3*, *Co-4*, and *Co-5* loci. Fine mapping of some of these resistance genes, *Co-x* [25]; *Co-1* [23]; *Co-1*^*2*^ [26] and the *Co-4*^*2*^ [27, 28] has been conducted and candidate genes associated with resistance identified. The *Co-x* gene was fine mapped to Pv01, adjacent to the *Co-1* locus [25] and fine mapping of the *Co-4* (*COK-4*) locus revealed 18 copies of the *COK-4* gene in a 325 kb segment of Pv08 [28]. Using this information, breeders have been developing anthracnose resistant cultivars for both subsistence and commercial producers [13]. However, the resistance offered by these cultivars tends to be ephemeral as they succumb to new races of anthracnose within a few years of their release, likely the result of extreme virulence polymorphism(s) in the pathogen population [11, 29]. Even with the availability of management practices, such as seed and foliar treatment with fungicides, crop rotation, the use of certified seed and genetic resistance, bean anthracnose continues to occur regularly in most bean production areas.

The molecular-genetic mechanisms underpinning the bean-anthracnose interaction are poorly understood, despite the mapping of numerous host resistance (R) genes and quantitative trait loci (QTL) controlling resistance [11, 30]. Differential gene expression between the compatible and incompatible interaction was reported by Fraire-Velazquez and Lozoya-Gloria [31], where the putative phenylalanine ammonia lyase (*PAL*) and chalcone synthase (*CHS*) genes were identified as up-regulated in resistant bean lines. As an extension of this initial work, subsequent work by Oblessuc et al. [32] used an EST library approach to uncover putative mechanisms associated with the activation of innate immunity in the SEL1308 bean genotype carrying the *Co-4*^*2*^ anthracnose R-gene. From this, it was observed that *PRb1a* and *PR1b* transcripts were induced after 72 and 96 hours post inoculation (hpi), irrespective of tissues (leaves, epicotyl and hypocotyl), in the incompatible interaction between the resistant genotype SEL 1308 and race 73 of *C*. *lindemuthianum*. Additional work has also shown that several defense-related genes are down-regulated [33]. In the *P*. *vulgaris*-*Colletotrichum* interaction, activation of the plant defense response depends on the recognition of avirulence determinants by plant receptors. Upon recognition, specific signal transduction pathways are triggered resulting in the production of reactive oxygen species (ROS) [34]; accumulation of pathogenesis-related (PR) proteins [33]; phytoanticipins [35]; enzymes derived from host and involved in secondary metabolism [36, 37, 38]; and localized cell death [39].

Whole transcriptome sequencing analysis during host infection by virulent and avirulent pathogens can provide insight into the molecular mechanisms involved in compatible and incompatible interactions. Among the various techniques used to delineate early responses in plant-pathogen interaction, whole transcriptome sequencing (RNA-seq) has been successfully conducted between various plant-pathogen interactions and comprehensive insights on early period of pathogenesis were revealed. For example, Li et al. [40] reported substantial number of banana transcripts regulated during banana and *Fusarium oxysporum* f. sp. *cubense* interface. Lowe et al. [41] used RNA-seq to delineate the mechanisms related to pathogenesis of *Brassica napus* from two related fungus species viz., *Leptosphaeria maculans* and *L*. *biglobosa*. Analysis of the DEGs showed different up and down regulated transcripts in both the interactions. An interaction between sugarcane and *Sporisorium scitamineum* revealed early expression of defense related genes in the resistant sugarcane genotype in comparison to the susceptible one [42] Although more examples of model as well as non-model plant-fungus interactions have been reported [43, 44, 45, 46, 47], there are only a few reports of differential gene expression between host plants and *Colletotrichum* species using RNA-seq. In total, these studies have led to a better understanding of the early defense signaling mechanisms during pathogen infection and proliferation. For example, Ludwig et al. [48] reported melanin as indispensable for pathogenesis in *C*. *graminicola*. Similarly, comparative transcriptome studies between *C*. *higginsianum—Arabidopsis thaliana* and *C*. *graminicola—Zea mays* pathosystem revealed genes related to pathogenicity determinants and effectors are up regulated during the biotrophic phase of pathogenesis whereas genes related to hydrolases and transporters are up regulated during the necrotrophic phase. Simultaneous transcriptome studies between tomato fruit and *C*. *gloeosporioides* at three different ripening stages has provided in-depth gene regulation during early and late phases of pathogenesis [49]. However, the available data are limited in various host-*Colletotrichum* interactions including the *P*. *vulgaris*-*C*. *lindemuthianum* anthracnose interface.

The focus of this study was to conduct a detailed transcriptomic analysis using RNA-seq analysis of the *P*. *vulgaris*-*C*. *lindemuthianum* pathosystem to discover genes–and their associated processes–that are regulated during the compatible and incompatible interactions and identify genes underlying pathogenicity and resistance in the *P*. *vulgaris- C*. *lindemuthianum* interaction.

## Materials and Methods

### Bean genotypes

A near isogenic (NIL) pair of bean genotypes was used in the present study. The NILs were developed from among three heterogeneous F_4:6_ recombinant inbred lines (RILs) [50] extracted from a 96-member RIL population derived from the cross of Jaguar and Puebla 152 black bean cultivars. Jaguar possess the *Co-1* gene that provides resistance to race 73 of *C*. *lindemuthianum* whereas Puebla 152 lacks the resistance allele [23, 51]. The final NIL pair selected (10T-9576) was similar in all phenotypic characters except in response to inoculation with race 73 of *C*. *lindemuthianum*. The NIL (T-9576R) with resistance to race 73 of *C*. *lindemuthianum* possessed the *Co-1* gene on chromosome Pv01, whereas the susceptible NIL (T-9576S) possessed the susceptible *co-1* allele. The phenotypic reaction of the NIL pair was confirmed by inoculation prior to the initiation of the study.

### Inoculation with Bean anthracnose race 73

Race 73 of *C*. *lindemuthianum* is the most widespread race and accounts for nearly 25% of the pathogen population in North America [16]. Because of the importance of race in Michigan it was used for delineating the early responses of bean-anthracnose interface. Seeds of the resistant and susceptible NIL pair (6 each) were planted in trays and maintained following the desired agronomic practices. Race 73 was maintained on Mathur media and 10 days old fungus was used to inoculate the plants. Conidia at a concentration of 2 x 10^6^ spores were used for the inoculation. Distilled water was sprayed on the control plants. To permit disease development, inoculated plants were maintained in a greenhouse at 22°C ± 2°C with 90% humidity and a 12 h photoperiod.

### Total RNA isolation, cDNA library construction, and sequencing

A total of 48 leaf samples were collected from both the resistant and susceptible NILs, including inoculated and mock-inoculated, at 0, 24, 72, and 96 hpi at trifoliate growth stage. Immediately after collection, samples were flash frozen in liquid nitrogen, and stored at -80°C prior to RNA extraction. TRIzol Kit (Invitrogen, Carlsbad, CA, USA) was used to extract total RNA, and contaminant DNA was removed using DNAase, following the manufacturer’s protocol. Total RNA concentration and purity was measured using a NanoDrop 2000 (Thermo Fisher Scientific, Waltham, MA, USA). RNA integrity was checked using a Biological analyzer Agilent 2100 (Agilent, Santa Clara, CA, USA), and Agilent RNA 6000 Pico kit, following the manufacturer’s protocol. The Michigan State University Research Technology Support Facility prepared a total of 48 mRNA-seq libraries using the Illumina TruSeq Stranded mRNA Library Preparation Kit following the manufacturer’s protocol. Libraries were pooled for multiplexed sequencing on an Illumina HiSeq 2500. Sequencing was performed using Illumina HiSeq SBS reagents to generate single end (SE) reads of 50 nucleotides (nt).

### Bioinformatics analyses

Read quality was assessed using FastQC [52]. Cutadapt v1.8.1 [53] was used to remove adapters from reads, and only reads with at least 30 nt (after removing adapters) were retained. Retained reads were aligned to the *Phaseolus vulgaris* v1.0 reference genome [54] using TopHat2 version 2.0.14 [55], allowing up to a maximum of two mismatches. The minimum and maximum intron size was set to 4 bp and 11,000 bp, respectively. All other parameters for TopHat were used at default settings. Cufflinks version 2.2.1 [56] was used to determine the expression status of the genes. For each gene, normalized gene expression level was computed as fragments per kilobase (kb) pair of exon model per million fragments mapped (FPKM). A gene was considered expressed if its FPKM 95% confidence interval lower boundary was greater than zero. The accepted BAM hits from alignments were converted to SAM format using SAMTools v0.1.18 [57]. The number of reads that mapped to a gene were counted using htseq-count from the HTSeq.py python package [58]. Normalized read count for each sample was computed by dividing the raw read count from htseq-count by the library size using DESeq2 R package [59].

### Identification of differentially expressed genes and enriched gene ontology terms

Differential gene expression analysis was performed using DESeq2, in R, based on normalized read count. A false discovery rate (FDR) < 0.01 (Benjamini–Hochberg correction) was used to determine differential gene expression significance. DEGs were filtered further for fold expression change, and only genes with absolute Log2 fold-change (|Log2FC|) ≥ 2 were retained for downstream analyses. DEGs between resistant and susceptible NIL were identified in three steps. First, DEGs between resistant and susceptible NILs (mock-inoculated) were identified at 0, 24, 72 and 96 hpi. Second, DEGs between resistant and susceptible NILs (inoculated) were identified at 0, 24, 72 and 96 hpi. Next, we subtracted the DEGs between resistant and susceptible of the mock-inoculated NILs from the DEGs of the inoculated at 0, 24, 72, and 96 hpi. The remaining DEGs at each time point after this subtraction step were considered to be informative, possibly functionally associated with balancing the resistance and susceptibility of the two NILs to *C*. *lindemuthianum* race 73.

Gene ontology (GO) term [60] enrichment analysis of DEGs (with |Log2FC| ≥ 2) between resistant and susceptible NILs was conducted. The singular enrichment analysis tool from AgriGO [61] was used with GO annotations from *P*. *vulgaris* v1.0 reference genome. Singular enrichment analysis was done using Fisher’s test, and a FDR cut-off of 0.05 was used to identify significantly enriched biological processes and molecular functions of the genes up-regulated in the resistant and susceptible NIL at 0, 24, 72, and 96 hpi.

### Identification of differentially expressed genes in the *Co-1*, *Co-x* and *Co-4* loci

The *Co-1*, *Co-x*, and *Co-4* resistant loci have been mapped to chromosomes Pv01, Pv01 and Pv08, respectively. The genomic regions to which these loci have been mapped remain large even in the case of *Co-1* gene (525 kb) that has been fine mapped. As a result, the large genomic regions for these loci have produced many positional candidate genes. To narrow down the number of positional candidate genes, the *Co-1*, *Co-x* and *Co-4* loci were searched for DEGs. These DEGs were considered as both positional and expression candidates, making their candidacy stronger. For the *Co-1* and *Co-x* loci we investigated a 400 kb region spanning 50.0–50.4 Mb on Pv01, which contains the genomic regions identified by Richards et al. [25] and Zuiderveen et al. [23] who mapped the *Co-x* and *Co-1* genes, respectively. In the case of the *Co-4* locus the 325 kb region on Pv08 proposed by Oblessuc et al. [28] was searched as the most probable region for *Co-4* gene.

## Results

### Disease development in the NIL pair

**Fig 1:**
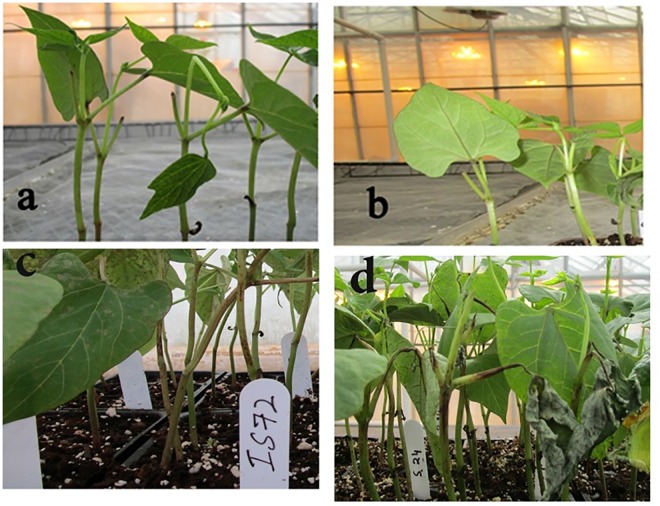
Disease progression on the susceptible NIL genotype a) 0 hpi b) 24 hpi c) 72 hpi and d) 96 hpi. Water soaked lesions were seen at 72 hpi and plants exhibited necrotic lesions at 96 hpi.

**Fig 2:**
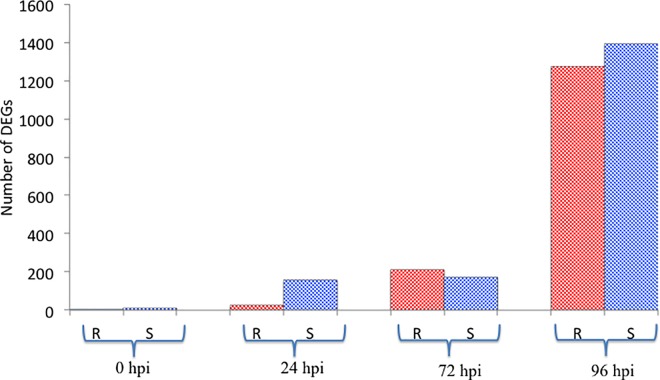
Number of genes up-regulated in resistant (R) and susceptible (S) near isogenic lines at 0, 24, 72, and 96 hours post inoculation with *Colletotrichum lindemuthianum* race 73.

Two NILs differing at *Co-1* locus were inoculated with *C*. *lindemuthianum* race 73 and disease development was observed. In the inoculated mock, resistant, and susceptible NIL pair, disease symptoms developed after 72 hpi (Fig 1). Prior to that time, we did not observe any differences between treatments. After 72 hpi, small water soaked lesions were observed on the veins on the adaxial surface of the leaves, as well as small lesions on the stem of the susceptible NIL. These water soaked lesions further developed into necrotic lesions at 96 hpi. The resistant NIL did not produce any symptoms or hypersensitive response even after 7 dai, whereas the inoculated susceptible NIL produced larger sporulating lesions both on leaves and stems (Fig 1D). Sunken lesions on the stem of the susceptible NIL resulted in plant death. These results confirm the hemibiotrophic nature of the fungus and that the biotrophic phase is active until 72 h, when the fungus switches to the necrotrophic phase after the colonized tissue is dead. In contrast, the immune response of the inoculated resistant NIL showed the elicitation of defense responses at an early stage of the interaction (Fig 2).

### Transcriptome analyses

A total of 48 RNA-seq libraries were made from leaf tissues of resistant and susceptible NILs collected at 0, 24, 72 and 96 hpi. A total of 1,940 M nt SE reads were generated. The number of reads per library ranged from 19.2 million (M) to 59.5 M, with an average of 40.4 M (S1 Table). The average mapping rate of each library to the reference genome was 84.2%. The average percentage of uniquely mapped reads was 89.2% of the total mapped reads for each library.

### Differentially expressed genes and enriched gene ontology terms

A total of 12 genes were differentially expressed between resistant and susceptible NIL at 0 hpi (Fig 2; S2 Table). Of the 12 DEGs, only one was up-regulated in the resistant NIL, while the other 11 were up-regulated in the susceptible NIL. There were no significantly enriched GO terms in DEGs at 0 hpi.

A total of 184 genes were differentially expressed between resistant and susceptible NILs at 24 hpi (Fig 2; S3 Table). Of the 184 DEGs, 26 were up-regulated in the resistant NIL while 158 were up-regulated in the susceptible NIL. Three biological processes including cell redox homeostasis (GO:0045454), lipid metabolic process (GO:0006629), iron ion binding (GO:0005506) were significantly enriched in genes up-regulated in the susceptible NIL at 24 hpi (Table 1). Enriched molecular functions of genes up-regulated in the susceptible NIL included hydrolase activity (GO:0016788), disulfide oxidoreductase activity (GO:0015035), and electron carrier activity (GO:0009055). There were no significantly enriched GO terms in genes up-regulated in the resistant NIL.

**Table 1:** Gene ontology terms enriched in genes differentially expressed between resistant and susceptible near isogenic lines at 24, 72 and 96 hours post inoculation with *Colletotrichum lindemuthianum* race 73.

GO term^a^	Description	P-value	FDR
**GO for genes down-regulated in resistant line at 24 hpi**			
GO:0045454 (P)	Cell redox homeostasis	3.4E-05	0.0011
GO:0006629 (P)	Lipid metabolic process	2.7E-06	0.0002
GO:0005506 (P)	Iron ion binding	0.0017	0.018
GO:0015035 (F)	Disulfide oxidoreductase activity	5.9E-06	0.0001
GO:0016788 (F)	Hydrolase activity	0.0004	0.0054
GO:0009055 (F)	Electron carrier activity	4.6E-06	0.0001
**GO for genes up-regulated in resistant line at 72 hpi**			
GO:0009767 (P)	Photosynthetic electron transport	1.0E-08	1.3E-06
**GO for genes up-regulated in resistant line at 96 hpi**			
GO:0009607 (P)	Response to biotic stimulus	6.0E-05	0.0045
GO:0043086 (P)	Regulation of catalytic activity	1.7E-05	0.0019
GO:0006073 (P)	Cellular glucan metabolic process	2.1E-05	0.0019
GO:0009765 (P)	Photosynthesis, light harvesting	1.2E-10	8.9E-08
GO:0016762 (F)	Transferase activity	1.7E-06	0.0003
GO:0004553 (F)	Hydrolase activity	2.6E-11	7.4E-09
GO:0042802 (F)	Identical protein binding	3.1E-05	0.0044
GO:0005507 (F)	Copper ion binding	8.5E-05	0.0081
**GO for genes down-regulated in resistant line at 96 hpi**			
GO:0006032 (P)	Chitin catabolic process	0.0004	0.04
GO:0043687 (P)	Post-translational protein modification	0.0004	0.041
GO:0006979 (P)	Response to oxidative stress	0.0003	0.04
GO:0055114 (P)	Oxidation reduction	1.6E-17	1.4E-14
GO:0016998 (P)	Cell wall macromolecule catabolic	0.0002	0.04
GO:0003700 (F)	Transcription factor activity	0.0016	0.031
GO:0004601 (F)	Peroxidase activity	0.0001	0.0049
GO:0004568 (F)	Chitinase activity	0.0004	0.013
GO:0020037 (F)	Heme binding	3.8E-08	5.6E-06
GO:0008061 (F)	Chitin binding	0.0001	0.0049
GO:0016491 (F)	Oxidoreductase activity	6.5E-18	4.7E-15
GO:0004672 (F)	Protein kinase activity	0.0011	0.024

^a^GO = Gene ontology; P = Biological Process; F = Molecular Function; FDR = false discovery ratio

**Fig 3:**
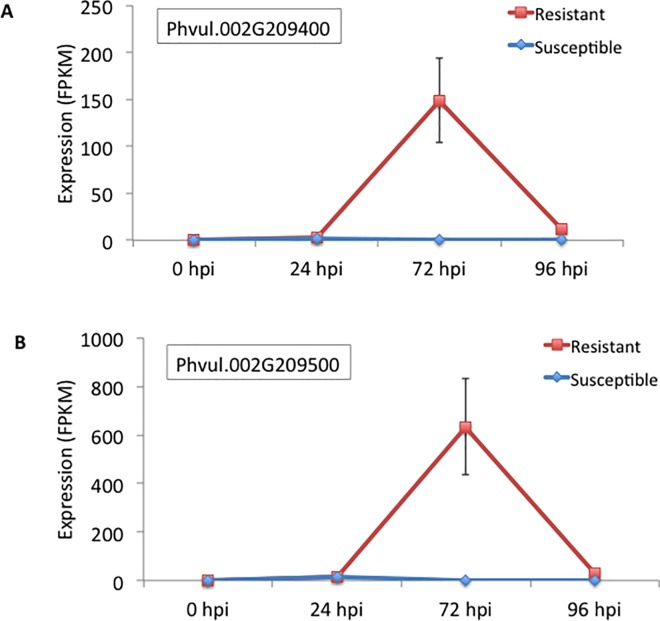
Expression patterns of Phvul.002G209400 (A) and Phvul.002G209500 (B) encoding pathogenesis-related proteins belonging to the Bet v 1 family in resistant and susceptible near isogenic lines inoculated with *Colletotrichum lindemuthianum* race 73.

A total of 385 genes were differentially expressed between resistant and susceptible NIL at 72 hpi (S4 Table). Of the 385 DEGs, 212 were up-regulated in the resistant NIL while 173 were up-regulated in the susceptible NIL (Fig 2). Among the 212 up-regulated genes in the resistant NIL, two genes with highest fold change expression Phvul.002G209500 (LogFC = 11) and Phvul.002G209400 (LogFC = 8) encode pathogenesis-related proteins belonging to the Bet v 1 family (Fig 3; S4 Table). Photosynthetic electron transport (GO:0009767) was the only enriched biological process up-regulated genes in the resistant NIL (Table 1). There were no significantly enriched GO terms among the 327 genes down-regulated in the resistant NIL at 72 hpi.

At 96 hpi 2669 genes were differentially expressed between resistant and susceptible NILs (S5 Table). Of the 2669 DEGs, 1,275 were up-regulated in the resistant while 1394 were up-regulated in the susceptible NIL (Fig 2). Phvul.002G209500 and Phvul.002G209400 that encode pathogenesis-related proteins, were strongly up-regulated in the resistant NIL at 72 hpi (Fig 3). Enriched biological processes of genes up-regulated in the resistant NIL included response to biotic stimulus (GO:0009607), regulation of catalytic activity (GO:0043086), cellular glucan metabolic process (GO:0006073), and photosynthesis (GO:0009765) (Table 1). Enriched molecular functions of genes up-regulated in the resistant NIL included hydrolase activity (GO:0004553), transferase activity (GO:0016762), identical protein binding (GO:0042802), and copper ion binding (GO:0005507) (Table 1). Enriched biological processes of genes up-regulated in the susceptible NIL included chitin catabolic process (GO:0006032), post-translational protein modification (GO:0043687), response to oxidative stress (GO:0006979), oxidation reduction (GO:0055114), and cell wall macromolecule catabolic (GO:0016998). The enriched molecular functions of up-regulated genes in the susceptible NIL included transcription factor activity (GO:0003700), peroxidase activity (GO:0004601), chitinase activity (GO:0004568), heme binding (GO:0020037), chitin binding (GO:0008061), oxidoreductase activity (GO:0016491), and protein kinase activity (GO:0004672).

### Differentially expressed genes encoding transcription factors

Transcription factors (TFs) have widely been reported to play important roles in plant defense and stress response. Thus, we were interested in identifying TFs that may be involved in regulating expression of genes involved in resistance to *C*. *lindemuthianum*. At 24 hpi, four TFs were differentially expressed, and all four were up-regulated in the susceptible NIL. Interestingly, all four encode WRKY TFs (Table 2). At 72 hpi, six genes encoding TFs were differentially expressed between resistant and susceptible NILs. These differentially expressed TFs belong to AP2, bZIP, Dof, Homeobox, and WRKY families. Of the six differentially expressed TF genes, three were up-regulated in resistant while three were up-regulated in susceptible NIL (Table 2). A total of 137 genes encoding TFs were differentially expressed between susceptible and resistant NILs at 96 hpi (Table 2). Of the 141 differentially expressed TFs, 37 were up-regulated in the resistant NIL while 104 up-regulated in the susceptible NIL. Differentially expressed TFs at 96 hpi belong to AP2 (17), bHLH (14), bZIP (1), Dof (4), ethylene-responsive (12), GATA (2), GT-2 (1), heat shock (10), homeobox (6), MYB (22), apical meristem (19), PLATZ (4), TCP (6), and WRKY (19) families (Table 2). Eighteen of the 19 differentially expressed WRKY TFs at 96 hpi were up-regulated in the susceptible NIL. Of the 19 differentially expressed apical meristem TFs at 96 hpi, 18 were up-regulated in the susceptible NIL, and only one was up-regulated in resistant NIL.

**Table 2:** Genes encoding transcription factors differentially expressed between resistant and susceptible near isogenic lines at 24, 72 and 96 hours post inoculation with *Colletotrichum lindemuthianum* race 73.

Gene ID	Log2FC^a^	Transcription Factor
**24 hpi**		
Phvul.009G137500	-2.1	WRKY
Phvul.002G196800	-2.1	WRKY
Phvul.010G062500	-2.2	WRKY
Phvul.003G124000	-2.4	WRKY
**72 hpi**		
Phvul.002G016700	2.1	AP2
Phvul.009G093600	-3.5	AP2
Phvul.007G025500	2.8	bZIP
Phvul.011G064800	-2.0	Dof
Phvul.009G049600	2.7	Homeobox
Phvul.001G042100	-2.2	WRKY
**96 hpi**		
Phvul.001G084000	-8.2	AP2
Phvul.002G146400	-2.8	AP2
Phvul.004G069900	-4.0	AP2
Phvul.006G106100	-3.5	AP2
Phvul.007G193300	-2.7	AP2
Phvul.007G193700	-4.4	AP2
Phvul.001G160500	-4.9	AP2
Phvul.002G267800	-3.7	AP2
Phvul.002G281300	-3.5	AP2
Phvul.002G310200	3.1	AP2
Phvul.003G223600	3.8	AP2
Phvul.003G232600	2.1	AP2
Phvul.005G183700	2.9	AP2
Phvul.006G179800	-2.6	AP2
Phvul.008G019600	-2.7	AP2
Phvul.008G253600	3.5	AP2
Phvul.009G137900	-2.9	AP2
Phvul.003G094700	-2.6	bHLH
Phvul.009G179600	-2.2	bHLH
Phvul.002G229100	2.4	bHLH
Phvul.004G027700	5.0	bHLH
Phvul.005G068500	3.2	bHLH
Phvul.005G097800	3.2	bHLH
Phvul.006G184600	2.2	bHLH
Phvul.007G194600	2.2	bHLH
Phvul.008G041800	4.7	bHLH
Phvul.006G196600	3.8	bHLH
Phvul.002G282300	-2.1	bHLH
Phvul.005G036900	-2.3	bHLH
Phvul.009G137400	-2.1	bHLH
Phvul.009G235700	-2.5	bHLH
Phvul.006G078500	2.0	bZIP
Phvul.006G188300	2.9	Dof
Phvul.002G226100	-2.1	Dof
Phvul.002G238400	-4.2	Dof
Phvul.006G176400	-5.0	Dof
Phvul.001G160100	-7.1	Ethylene-responsive TF
Phvul.007G127800	-4.2	Ethylene-responsive TF
Phvul.007G193400	-3.7	Ethylene-responsive TF
Phvul.007G193900	-5.0	Ethylene-responsive TF
Phvul.007G272800	-7.7	Ethylene-responsive TF
Phvul.007G273000	-4.8	Ethylene-responsive TF
Phvul.008G098900	-2.7	Ethylene-responsive TF
Phvul.003G241700	2.8	Ethylene-responsive TF
Phvul.004G092100	-8.3	Ethylene-responsive TF
Phvul.007G128100	-3.4	Ethylene-responsive TF
Phvul.009G089300	3.7	Ethylene-responsive TF
Phvul.010G114900	2.1	Ethylene-responsive TF
Phvul.004G079200	2.5	GATA
Phvul.010G146300	2.3	GATA
Phvul.011G205900	2.2	GT-2
Phvul.001G131000	-6.8	Heat Shock TF
Phvul.002G228400	-8.8	Heat Shock TF
Phvul.008G202500	3.0	Heat Shock TF
Phvul.003G244000	-3.7	Heat Shock TF
Phvul.006G034200	-2.1	Heat Shock TF
Phvul.009G065800	2.7	Heat Shock TF
Phvul.001G266300	-3.0	Heat Shock TF
Phvul.002G019100	-5.4	Heat Shock TF
Phvul.007G067800	-3.4	Heat Shock TF
Phvul.004G087500	-2.7	Heat Shock TF
Phvul.002G282300	-2.1	bHLH
Phvul.005G036900	-2.3	bHLH
Phvul.009G137400	-2.1	bHLH
Phvul.009G235700	-2.5	bHLH
Phvul.001G224900	-2.9	Homeobox
Phvul.002G056300	2.6	Homeobox
Phvul.002G094100	-2.8	Homeobox
Phvul.003G138200	-3.7	Homeobox
Phvul.006G116000	-5.1	Homeobox
Phvul.010G117200	-4.0	Homeobox
Phvul.004G171200	3.7	MYB
Phvul.001G019200	-3.8	MYB
Phvul.001G179400	-2.7	MYB
Phvul.002G184600	-3.1	MYB
Phvul.002G306000	-3.2	MYB
Phvul.002G317000	-7.5	MYB
Phvul.003G094600	-5.5	MYB
Phvul.005G141200	2.7	MYB
Phvul.006G025400	-7.7	MYB
Phvul.006G114800	-7.1	MYB
Phvul.007G108500	-5.2	MYB
Phvul.007G211900	-3.7	MYB
Phvul.007G273400	-3.7	MYB
Phvul.009G075000	-4.0	MYB
Phvul.010G079300	2.3	MYB
Phvul.010G115500	-5.6	MYB
Phvul.011G084500	-4.1	MYB
Phvul.008G098300	-2.9	MYB
Phvul.004G057800	2.6	MYB
Phvul.004G116500	-4.6	MYB
Phvul.005G123900	2.0	MYB
Phvul.006G120800	-4.4	MYB
Phvul.001G072200	-2.6	No apical meristem (NAM)
Phvul.001G100500	-3.7	No apical meristem (NAM)
Phvul.002G275000	-2.7	No apical meristem (NAM)
Phvul.004G076900	-4.3	No apical meristem (NAM)
Phvul.005G055400	-2.5	No apical meristem (NAM)
Phvul.005G084500	-4.0	No apical meristem (NAM)
Phvul.008G194600	-2.6	No apical meristem (NAM)
Phvul.011G095500	-2.4	No apical meristem (NAM)
Phvul.001G100200	-3.1	No apical meristem (NAM)
Phvul.002G170200	-4.5	No apical meristem (NAM)
Phvul.003G045600	-2.9	No apical meristem (NAM)
Phvul.003G189000	-2.8	No apical meristem (NAM)
Phvul.004G028300	-3.8	No apical meristem (NAM)
Phvul.004G029900	-3.9	No apical meristem (NAM)
Phvul.004G077400	-4.1	No apical meristem (NAM)
Phvul.009G156300	-3.2	No apical meristem (NAM)
Phvul.009G163200	2.5	No apical meristem (NAM)
Phvul.011G147800	-4.6	No apical meristem (NAM)
Phvul.008G001000	-2.9	No apical meristem (NAM)
Phvul.001G183400	3.0	PLATZ
Phvul.003G284800	-2.2	PLATZ
Phvul.007G226200	2.0	PLATZ
Phvul.008G142900	-3.9	PLATZ
Phvul.002G086600	2.1	TCP
Phvul.003G190900	2.2	TCP
Phvul.003G213300	-2.9	TCP
Phvul.004G037700	-3.0	TCP
Phvul.009G188100	2.2	TCP
Phvul.011G156900	2.5	TCP
Phvul.001G214400	-3.7	WRKY
Phvul.002G196800	-2.7	WRKY
Phvul.002G285800	-8.6	WRKY
Phvul.003G124000	3.5	WRKY
Phvul.003G192000	-3.6	WRKY
Phvul.006G147800	-3.1	WRKY
Phvul.007G209000	-3.5	WRKY
Phvul.007G212900	-2.9	WRKY
Phvul.008G090300	-2.8	WRKY
Phvul.009G189700	-5.9	WRKY
Phvul.001G042100	-5.3	WRKY
Phvul.001G042200	-3.1	WRKY
Phvul.001G088200	-5.4	WRKY
Phvul.002G160100	-5.5	WRKY
Phvul.002G265400	-3.9	WRKY
Phvul.006G074600	-4.4	WRKY
Phvul.009G080000	-5.6	WRKY
Phvul.009G101900	-2.1	WRKY
Phvul.011G101900	-2.7	WRKY

^a^Log2FC = Logarithmic fold change of expression (resistant/susceptible NIL), where positive is up-regulated and negative is down-regulated in resistant NIL

### Differentially expressed genes encoding NB-ARC and LRR-Receptor domains

**Fig 4:**
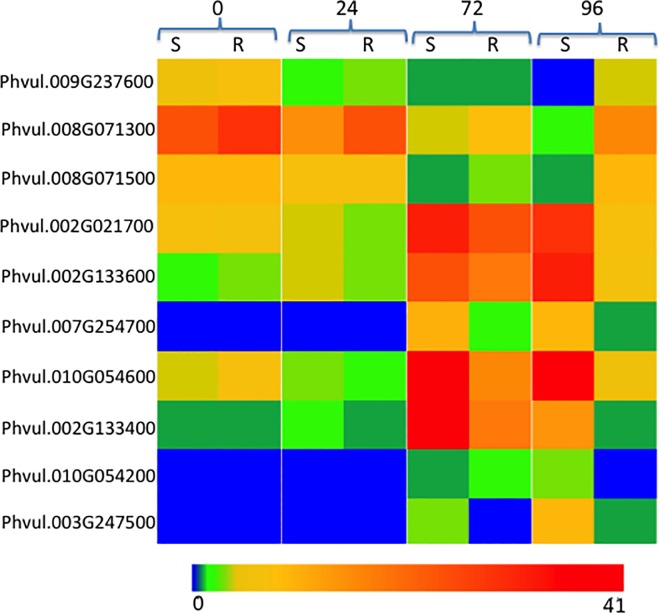
Heatmap of expression (FPKM) for nine genes encoding NB-ARC domains that were differentially expressed between resistant and susceptible near isogenic lines at 0, 24, 72 and 96 hours post inoculation with *Colletotrichum lindemuthianum* race 73. Expression for these nine genes ranged from 0 to 41 FPKM.

Resistance genes in plants encode proteins that recognize pathogen attack and activate innate immune response. These proteins contain a central nucleotide-biding domain called NB-ARC, which is believed to regulate their activity. Given the importance of NB-ARC proteins, we were interested in identifying DEGs that encode proteins with the NB-ARC domains. At 72 hpi, only one gene encoding a NB-ARC domain-containing protein was differentially expressed, and was up-regulated in the susceptible NIL (Table 3). At 96 hpi, 10 genes encoding NB-ARC domain-containing proteins were differentially expressed, and of these, 3 were up-regulated in the resistant NIL, while 7 were up-regulated in the susceptible NIL (Fig 4). Interestingly, there were no DEGs encoding NB-ARC domain-containing proteins identified at 0 and 24 hpi.

**Table 3:** Genes encoding NB-ARC and leucine-rich repeat domains that were differentially expressed between resistant and susceptible near isogenic lines at 72 and 96 hours post inoculation with *Colletotrichum lindemuthianum* race 73.

Gene ID	Domain	Log2FC^a^
**72 hpi**^b^		
Phvul.003G247500	NB-ARC	-2.1
Phvul.011G194100	LRR^c^	2.3
Phvul.001G243700	LRR	2.9
Phvul.004G099900	LRR	-2.7
Phvul.004G100100	LRR	-2.1
Phvul.004G103000	LRR	-2.1
Phvul.005G077700	LRR	-2.6
Phvul.005G162600	LRR	-2
**96 hpi**		
Phvul.009G237600	NB-ARC	3
Phvul.008G071300	NB-ARC	2.7
Phvul.008G071500	NB-ARC	2.4
Phvul.002G021700	NB-ARC	-2.1
Phvul.002G133600	NB-ARC	-2.4
Phvul.007G254700	NB-ARC	-2.5
Phvul.010G054600	NB-ARC	-2.8
Phvul.002G133400	NB-ARC	-3.2
Phvul.010G054200	NB-ARC	-3.3
Phvul.003G247500	NB-ARC	-5.7
Phvul.003G193100	LRR	4.1
Phvul.004G149100	LRR	3.8
Phvul.007G112100	LRR	3.5
Phvul.003G029700	LRR	3.3
Phvul.005G106800	LRR	3.1
Phvul.006G080200	LRR	3.1
Phvul.001G185400	LRR	3.1
Phvul.001G134600	LRR	3
Phvul.002G295600	LRR	3
Phvul.002G007800	LRR	2.9
Phvul.008G122200	LRR	2.8
Phvul.010G064300	LRR	2.8
Phvul.011G007200	LRR	2.7
Phvul.006G146100	LRR	2.7
Phvul.007G040400	LRR	2.7
Phvul.004G128100	LRR	2.6
Phvul.009G067200	LRR	2.6
Phvul.002G242600	LRR	2.5
Phvul.006G174100	LRR	2.5
Phvul.004G046800	LRR	2.4
Phvul.008G080500	LRR	2.3
Phvul.004G086300	LRR	2.2
Phvul.009G260900	LRR	2.2
Phvul.009G114400	LRR	2.1
Phvul.003G015800	LRR	2.1
Phvul.005G120300	LRR	2.1
Phvul.008G044600	LRR	-2.3
Phvul.005G162000	LRR	-2.3
Phvul.004G175900	LRR	-2.4
Phvul.006G198200	LRR	-2.4
Phvul.008G146000	LRR	-2.5
Phvul.008G044700	LRR	-2.5
Phvul.007G137800	LRR	-2.8
Phvul.009G112200	LRR	-2.8
Phvul.004G115600	LRR	-2.9
Phvul.008G079800	LRR	-2.9
Phvul.004G136500	LRR	-3
Phvul.004G175800	LRR	-3.2
Phvul.006G180200	LRR	-3.3
Phvul.009G184500	LRR	-3.9
Phvul.009G046900	LRR	-4.7
Phvul.003G187200	LRR	-4.9
Phvul.008G043400	LRR	-6

^a^Log2FC = Logarithmic fold change of expression (resistant/susceptible NIL), where positive is up-regulated and negative is down-regulated in resistant NIL

^b^72 and 96 hpi = 72 and 96 hours post inoculation

^c^LRR = leucine-rich repeat

To investigate which host R-genes themselves were differentially regulated, we examined the expression profiles of leucine-rich repeat (LRR)-encoding genes, as many of these proteins belong to the family of disease R proteins. As shown in Table 3, at 72 hpi, 7 genes encoding LRR domains were differentially expressed, with 2 up-regulated in the resistant NIL while 5 were up-regulated in the susceptible NIL. At 96 hpi, 43 genes encoding LRR domains were differentially expressed; of these, 26 were up-regulated in the resistant NIL while 17 were up-regulated in the susceptible NIL.

### Differentially expressed genes in *Co-1*, *Co-x* and *Co-4* genomic regions

**Fig 5:**
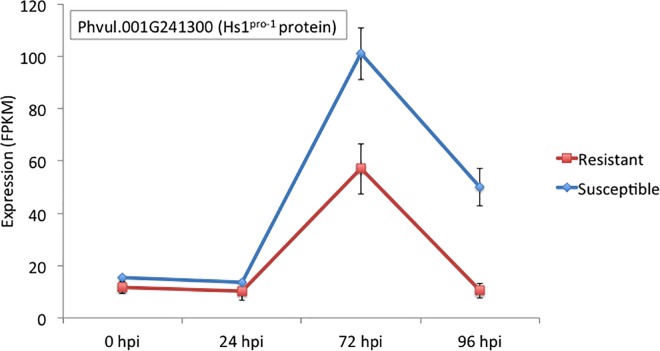
Expression patterns of Phvul.001G241300 that encoding a Hs1pro-1 protein in resistant and susceptible near isogenic lines inoculated with *Colletotrichum lindemuthianum* race 73.

A total of 48 genes were identified in the genomic region 50.0 Mb-50.4 Mb reported to contain *Co-1* and *Co-x* loci. Of these 48 genes, only six were differentially expressed between resistant and susceptible NILs (Table 4). These 6 include Phvul.001G242400 (encoding a putative hydrolase), Phvul.001G243500 (encoding a putative tyrosine kinase), and Phvul.001G244000 (encoding a putative oxygenase) that were up-regulated in the susceptible NIL at 24, 72 and 96 hpi, respectively. The fourth DEG was Phvul.001G243700, which encodes a putative kinase, and was up-regulated in the resistant NIL at both 72 and 96 hpi. The fifth DEG was Phvul.001G241300 that encodes an Hs1^pro-1^ protein (Fig 5). This is interesting, as in sugar beet (*Beta vulgaris* L.), Hs1^pro-1^ has been demonstrated to confer resistance to the beet cyst nematode *Heterodera schachtii* through a gene-for-gene mechanism [62]. The sixth DEG was Phvul.001G242200 that encodes Cytochrome P450, that was up-regulated in resistant at 96 hpi. The probable genomic region of the *Co-4* locus was recently reported to be a 325 kb region (2,245–2,570 kb) on Pv08. Of the 49 genes that are in this region, six were differentially expressed between resistant and susceptible NIL, and all were detected at 96 hpi (Table 4). These six DEGs include Phvul.008G029800 (encoding a putative kinase), Phvul.008G030200 (encoding a putative tyrosine kinase), Phvul.008G030300 (not annotated), and Phvul.008G030900 (not annotated) that were up-regulated in the resistant NIL. The other two were Phvul.008G028900 (glycosylated polypeptide) and Phvul.008G029300 (mitochondrial carrier) that were up-regulated in the susceptible NIL.

**Table 4:** Genes in *Co-1* and *Co-4* genomic regions that were differentially expressed between resistant and susceptible near isogenic lines at 24, 72 and 96 hours post inoculation with *Colletotrichum lindemuthianum* race 73.

Locus	Genomic region	Reference	Annotation	Hpi^a^	Differentially expressed gene	Log2FC^b^
Co-x	Pv01: 50.26–50.32 Mb	Richard et al. [25]	Cytochrome P450	96	Phvul.001G242200	2.1
Co-1	Pv01: 50.30 Mb	Zuiderveen et al. [23]	Kinase	96	Phvul.001G243700	5.2
			Hs1^pro-1^ protein	96	Phvul.001G241300	-2.1
			2OG-Fe(II) oxygenase	96	Phvul.001G244000	-2.2
			Kinase	72	Phvul.001G243700	2.9
			Tyrosine Kinase	72	Phvul.001G243500	-2.2
			Hydrolase	24	Phvul.001G242400	-2.1
Co-4	Pv08: 2.25–2.57 Mb	Oblessuc et al. [28]	Kinase	96	Phvul.008G029800	2.4
			Tyrosine Kinase	96	Phvul.008G030200	2.3
			Not annotated	96	Phvul.008G030300	4.3
			Not annotated	96	Phvul.008G030900	3.7
			Glycosylated polypeptide	96	Phvul.008G028900	-2.1
			Mitochondrial carrier	96	Phvul.008G029300	-2.1

^a^hpi = hours post inoculation

^b^Log2FC = Logarithmic fold change of expression (resistant/susceptible NIL), where positive is up-regulated and negative is down-regulated in resistant NIL

## Discussion

In the current study, the analysis of induced resistance and susceptibility responses following pathogen infection of resistant and susceptible NILs when challenged with anthracnose race 73 revealed alterations in many key biological and molecular functions. Understanding the mechanism employed by *C*. *lindemuthianum* to infect *P*. *vulgaris* and cause disease in the compatible interaction is crucial for disease management. The current transcriptomic analysis revealed the different mechanisms employed by the resistant and susceptible bean genotypes following infection by identifying which genes were expressed specifically at the early phase of pathogenesis.

### Disease development

In the present investigation, two NIL genotypes (T-9576S and T-9576R) differing at the *Co-1* locus were inoculated with bean anthracnose race 73. Disease progression observed visually showed no difference between resistant and susceptible NILs, except the water soaked lesions on leaves and stem of susceptible NIL at 72 hpi. After 96 hpi the susceptible NIL exhibited necrotrophy which clearly shows the hemibiotrophic nature of *C*. *lindemuthianum*. The biotrophic phase in the beginning of infection cycle is crucial for disease development. During this time period the fungus obtains nutrients from living tissues through the development of specialized infection structures such as appressorium, vesicles and primary hyphae. In contrast, during the necrotrophic phase, there is a breakdown of sugars by various enzymes. Our results clearly show expression of more genes in susceptible NIL than resistant during biotrophic phase (Fig 2). Many genes belonging to the sugar transporter (Phvul.007G088200, Phvul.002G046800), lipoxygenase (Phvul.010G135700) and peroxidase (Phvul.008G086900, Phvul.011G128200, Phvul.009G140700) were up regulated at 24 hpi. These genes are necessary for the defense response, whereas at 72 hpi most of these genes were down-regulated, which probably resulted in the development of disease and eventual necrotrophy. Similar to the data presented herein, the differential expression of sugar transporters during the necrotrophic phase have also been reported to be crucial for pathogenesis [63, 64, 65, 66].

### Defense related differentially expressed genes in compatible and incompatible interaction

RNA-seq, used to delineate the compatible and incompatible interaction between *P*. *vulgaris*—*C*. *lindemuthianum* (race 73) over a time course, revealed large differences in the mapping reads to the reference bean genome. At all-time points, mock inoculated resistant and susceptible NIL reads showed high percent mapping to the bean genome in comparison to the inoculated susceptible NIL at 72 and 96 hpi (S1 Table). More than 90% of reads uniquely mapped to the bean genome in the susceptible NIL at 0 and 24 hpi. However, at 96 hpi only 86% per cent reads mapped to the bean genome that clearly shows the fungus colonized the tissue (Fig 1) and some of the reads were of fungal nature. These reads need further investigation to identify the secretome of the fungus.

Many defense related genes were up-regulated during the time course of pathogenesis in the resistant NIL. These genes belonged to PR proteins (7 DEGs), chitinase (9 DEGs) peroxidases (3 DEGS), lipoxygenases (4 DEGs) whereas some of the genes, especially the sugar transporter, were up-regulated in susceptible NIL (S2, S3 and S4 Tables). Importance of defense related genes especially for cell wall reinforcement, and enzymes in the phenylpropanoid pathway are well established in bean/anthracnose pathosystem [33, 37]. These results suggest that the early expression of these genes in the resistant genotype may be crucial for disease suppression, as these transcripts were down-regulated in the susceptible interaction. Similarly, the up-regulation of sugar transporter genes after 72 hpi would indicate that the fungus needs various sugars for its survival and the up regulation of sugar transporters might be helping the fungus. At this phase various sugar breaking enzymes have been reported to be crucial for the survival of fungus in the necrotic lesion [67, 68, 69]. To cope with less carbon, the up regulation of genes for sugar transporters which have different substrate specificities represents a key factor in the development of the pathogen within its host [63, 65]. Host driven restrictions of nutritional compounds might be significant contributors of disease resistance in the resistant NIL. Since many defense-encoded transcripts were up-regulated in the resistant NIL, it appears that the resistant NIL recognizes the pathogen attack at an earlier stage of pathogenesis than the susceptible NIL. A similar conclusion was reported in transcriptome analysis of the cucumber /downy mildew interaction where large-scale changes in transcription occurred more rapidly in the resistant genotype [43].

### Differentially expressed transcriptional factors

Many genes that encode for TFs regulate the interaction between host and pathogen; this differential regulation is observed is both resistant and susceptible interactions. In the current study, we observed that four WRKY TFs were up-regulated in the susceptible NIL, with a pattern of increasing expression in the susceptible interaction over the time course of infection. This is potentially interesting, as WRKY TFs are known to regulate many key biological functions in the plant cell including biotic stress. Indeed, several WRKY TFs have been reported to positively regulate the activation of presumed defense genes during pathogen infection [70, 71, 72, 73]. However, some WRKY TFs have been reported as negative regulators of defense signaling thus enhancing the susceptibility in the host [74, 75, 76, 77]. Recently Wang et al. [78] conducted a genome wide analysis of WRKY TFs in common bean and found tissue specific expression of WRKY TFs. The WRKY TFs were distributed across all bean chromosomes and most of them were expressed in the roots but no TFs were expressed in the leaves over different time courses. The up regulated WRKY TFs during early pathogenesis may be responsible for susceptibility. We also observed two bZIP TFs up regulated in resistant NIL at 72 and 96 hpi. The bZIPs are the most studied TFs in plants [79] and their role in regulating various metabolic functions such as storage, leaf development, flower development and synthesis of hormones have been investigated [80, 81, 82, 83, 84, 85]. In addition to other functions, bZIP TFs have recently been reported to enhance disease resistance by activating salicylic acid (SA) dependent pathway. They also have been reported to interact with non-expresser of pathogen-related genes (NPR1) which is an important component of the SA defense response [86, 87, 88]. The role of various signaling hormones, such as SA and ethylene, in disease resistance activation is well documented [89, 90]. Most of the AP2 and ethylene responsive TFs were up regulated in the susceptible NIL. This clearly demonstrates that the resistance response due to PTI is suppressed in the susceptible NIL as these TFs play an important role in SA and jasmonic acid (JA) mediated pathways that contribute to acquired resistance.

### Differentially expressed resistance genes and candidates for *Co-1*, *Co-x* and *Co-4*

At 0 and 24 hpi, no plant R-gene candidate mRNAs were expressed, yet many were up-regulated both in the resistant and susceptible at 72 and 96 hpi. The bean-anthracnose interface displays gene-for-gene interaction and more than 20 anthracnose resistance genes have been identified in different genotypes [12]. The resistant NIL in the present investigation possesses the *Co-1* gene that was recently fine-mapped to the distal end of Pv01 [23]. A second gene, *Co-x* that resides in the vicinity of the *Co-1* was also fine mapped and reported to contain eight putative genes [25]. Among the eight candidate genes identified within the 58 kb region of the *Co-x*, our findings revealed that only two kinases and one cytochrome P450 were up-regulated (Table 4). These findings suggest that only three candidate genes are active within this region during pathogenesis. Up-regulation of resistance genes on Pv01 containing LRR and NB-ARC domains particularly Hs1^Pro-1^ (Phvul.001G241300) strongly favors the interaction of *C*. *lindemuthianum* effector with these genes. Hs1^Pro-1^ contains LRR and putative transmembrane domain [91] and is known to impart resistance to cyst nematodes in sugar beet on a gene-for gene basis. This is particularly interesting because the region around the *Co-x* and *Co-1* genes on Pv01 is syntenic with a region on chromosome Gm18 in soybean that possesses a major gene, *Rhg1* that conditions resistance to soybean cyst nematode from the same *Heterodera* genus [25]. There are many reports of dual nature of resistance imparted by plant resistance genes for example in tomato nematode resistance gene *Mi* also confers resistance to aphids [92]. Our observation of the differential expression of Hs1^Pro-1^ in the present investigation provides *prima facie* evidence that this gene may play a role in anthracnose, in addition to its described role in nematode resistance. Additionally, it is interesting to speculate that the upregulation of Hs1^Pro-1^ in the susceptible interaction is the result of the host compensating for destabilization of resistance during pathogen infection. Indeed, several reports have shown that HSP-type proteins are required for R-protein stabilization and activity during infection [93, 94]. However, further investigation is necessary to test this hypothesis.

Among the 49 candidate genes identified within a 325 kb region of the *Co-4* gene on Pv08 [28] only 4 genes were up regulated in the resistant NIL whereas one candidate gene (Phvul.008G028900) that encodes a glycosylated polypeptide was down regulated. Among the four candidate genes, one encode for a kinase and two are unannotated. The kinases at the *Co-4* locus have been reported to provide PTI in common bean [28]. Burt et al. [27] hypothesized that the presence of unique genes in addition to LRR and kinase proteins at the *Co-4* gene cluster may be imparting resistance. The hypothesis seems to be correct as the transcriptomic data from present investigation revealed two unannotated genes.

In the present investigation many candidate genes particularly kinases reported with the genomic region of *Co-1*, *Co-x* and *Co-4* genes were up regulated at 72 and 96 hpi. These kinases belong to receptor like kinase family [25, 27, 28, 95]. Receptor kinases play significant role in the recognition, signal transduction that ultimately lead to disease resistance [25, 27, 28, 96, 97, 98, 99]. The majority of cloned plant disease resistance genes encode proteins such as NBS, LRR, STK that are responsible for perception of avirulence proteins either directly or indirectly. However, the *Co-1* locus located at the distal end of Pv01 does not contain the typical NBS-LRR signature, and the candidate genes present at this locus are responsible for perception and signal transduction [25]. As a consequence of its atypical R gene nature these candidate genes up-regulate after biotrophy. We may have observed up-regulation of these candidate genes during the early bean-anthracnose interaction, had this region contained the typical R gene signatures that are responsible for perception of fungal effectors either directly or indirectly during the early phase of the infection process. To date, no resistance gene against bean anthracnose has been fully characterized, but few of them have been mapped and candidate genes identified. These candidates contain protein domains that are responsible for perception and signal transduction [13, 25, 28, 95]. Hence, PTI might be responsible for disease resistance in the resistant NIL. This hypothesis is based on the differential gene expression data that reflects activation of many transcriptional factors that are involved in PTI and defense related genes during the early phase of pathogenesis.

## Conclusions

In the present investigation, transcriptome profiling provided a dynamic picture of disease resistance and susceptibility in bean-anthracnose pathosystem that can be explained on the basis of gene expression. A total of 3,250 DEGs were identified between the NILs over the time course of infection. During the biotrophic phase of the interaction, the majority of DEGs were up-regulated in the susceptible NIL, whereas more DEGs were up-regulated in the resistant NIL during the necrotrophic phase. Various defense related genes such as PR proteins, peroxidases, lipoxygenases were up regulated in resistant NIL. Genes encoding sugar transporters were up-regulated in susceptible NIL during later phase of pathogenesis. Overall, the transcriptome analysis provided information that will facilitate a better understanding of the complex resistance phenomena between bean-anthracnose interactions. The strong up regulation of two PR proteins from the present investigation if over expressed in susceptible bean cultivars may provide additional anthracnose resistance. The Hs1^Pro-1^ resistance gene for beet cyst nematode was differentially expressed in resistant NIL and might be responsible for effector triggered immunity in beans. Many transcriptional factors and candidate genes within the vicinity of *Co-1*, *Co-x* and *Co-4* loci were differentially expressed. We were able to reduce the number of candidate genes reported at the *Co-1* and *Co-x* loci from eight to three and among the 49 candidate genes reported at *Co-4* locus only four were up regulated in the resistant NIL. Understanding the complexity of disease resistance should help in the development of bean cultivars that can better withstand the high virulence spectrum displayed by *C*. *lindemuthianum*.

## Supporting Information

S1 Table Statistics summary of read mapping to the common bean genome. ^a^Rep = replication; WS = susceptible near isogenic line mock inoculated with water; IS = susceptible near isogenic line inoculated with *Colletotrichum lindemuthianum* race 73; WR = resistant near isogenic line mock inoculated with water; IR = resistant near isogenic line inoculated with *Colletotrichum lindemuthianum* race 73; hpi = hours post inoculation.
(DOCX)

S2 Table List of genes that were differentially expressed between resistant and susceptible near isogenic line at 0 hour post inoculation with *Colletotrichum lindemuthiunum* race 73 (FDR = 0.05 and absolute Log2 fold change in expression greater than 2; NA means there is no functional annotation on Phytozome 10.3). (XLSX)

S3 Table List of genes that were differentially expressed between resistant and susceptible near isogenic line at 24 hours post inoculation with *Colletotrichum lindemuthiunum* race 73 (FDR = 0.05 and absolute Log2 fold change in expression greater than 2; NA means there is no functional annotation on Phytozome 10.3). (XLSX)

S4 Table List of genes that were differentially expressed between resistant and susceptible near isogenic line at 72 hours post inoculation with *Colletotrichum lindemuthiunum* race 73 (FDR = 0.05 and absolute Log2 fold change in expression greater than 2; NA means there is no functional annotation on Phytozome 10.3). (XLSX)

S5 Table List of genes that were differentially expressed between resistant and susceptible near isogenic line at 96 hours post inoculation with *Colletotrichum lindemuthiunum* race 73 (FDR = 0.05 and absolute Log2 fold change in expression greater than 2; NA means there is no functional annotation on Phytozome 10.3). (XLSX)
