# Supplementary material for: Transcriptome Profiling of the Phaseolus vulgaris - Colletotrichum lindemuthianum Pathosystem
Source: PLoS One. 2016 Nov 9;11(11):e0165823. doi: 10.1371/journal.pone.0165823 (PMC5102369; doi:10.1371/journal.pone.0165823)
Supplement: S1 Table — aRep = replication; WS = susceptible near isogenic line mock inoculated with water; IS = susceptible near isogenic line inoculated with Colletotrichum lindemuthianum race 73; WR = resistant near isogenic line mock inoculated with water; IR = resistant near isogenic line inoculated with Colletotrichum lindemuthianum race 73; hpi = hours post inoculation. (DOCX) [file pone.0165823.s001.docx]

S1 Table. Statistics summary of read mapping to the common bean genome

| Sample (Replication) | Total Reads | High-quality | Mapped (%) | Uniquely Mapped  (% of mapped) |
| --- | --- | --- | --- | --- |
| **0 hpi** |  |  |  |  |
| WS_Rep1^a^ | 36239519 | 36105970 | 29807362 (82.6%) | 27591570 (92.6%) |
| WS_Rep2 | 42986296 | 42923512 | 35355944 (82.4%) | 302344664 (85.5%) |
| WS_Rep3 | 43102996 | 43023258 | 33358412 (77.5%) | 29246666 (87.7%) |
| WR_Rep1 | 42202141 | 42153439 | 33802910 (80.2%) | 28475487 (84.2%) |
| WR_Rep2 | 39742416 | 39637465 | 32414678 (81.8%) | 29324550 (90.5%) |
| WR_Rep3 | 34562350 | 34432934 | 29166828 (84.7% ) | 26918779 (92.3%) |
| IS_Rep1 | 42860257 | 42752344 | 31802830 (74.4%) | 28263327(88.87%) |
| IS_Rep2 | 46014219 | 45740681 | 34971224 (76.5%) | 30820487(88.14%) |
| IS_Rep3 | 39715206 | 39684949 | 33274815 (83.8%) | 29863406 (89.7%) |
| IR_Rep1 | 41292412 | 41238202 | 34117639 (82.7%) | 29107160 (85.3%) |
| IR_Rep2 | 38400104 | 38368361 | 33265572 (86.7%) | 29856056 (89.8%) |
| IR_Rep3 | 45513094 | 45459093 | 37395196 (82.3%) | 32851606 (87.8%) |
| **24 hpi** |  |  |  |  |
| WS_Rep1 | 43677619 | 43628479 | 38638911 (88.6%) | 35763095 (92.6%) |
| WS_Rep2 | 37145818 | 37087458 | 33572843 (90.5% | 31216280 (93.0%) |
| WS_Rep3 | 46931381 | 46865550 | 41407902 (88.4%) | 37808836 (91.3%) |
| WR_Rep1 | 42597844 | 42495686 | 37341169 (87.9%) | 33801143 (90.5%) |
| WR_Rep2 | 21044156 | 21039965 | 18016843 (85.6%) | 16397935 (91.0%) |
| WR_Rep3 | 28316719 | 28312260 | 24002560 (84.8%) | 20903679 (87.1%) |
| IS_Rep1 | 26542420 | 26536948 | 20380669 (76.8%) | 17138169 (84.1%) |
| IS_Rep2 | 23072891 | 23069440 | 17978288 (77.9%) | 7438906 (87.8%) |
| IS_Rep3 | 28006241 | 28004411 | 25214406 (90.0%) | 23602571 (93.6%) |
| IR_Rep1 | 19219933 | 19218601 | 14721931 (76.6%) | 12228816 (83.1%) |
| IR_Rep2 | 20757779 | 20756739 | 16863713 (81.2%) | 15169087 (89.9%) |
| IR_Rep3 | 20637465 | 20635800 | 16986695 (82.3%) | 15271898 (89.9%) |
| **72 hpi** |  |  |  |  |
| WS_Rep1 | 44676801 | 44591845 | 39184171 (87.9%) | 36428016 (93.0%) |
| WS_Rep2 | 41167903 | 41132562 | 36794676 (89.5%) | 34145311 (92.8%) |
| WS_Rep3 | 50684646 | 50653465 | 39814624 (78.6%) | 32953313 (82.8%) |
| WR_Rep1 | 48086949 | 48051644 | 41850839 (87.1%) | 38200474 (91.3%) |
| WR_Rep2 | 43521131 | 43470712 | 34902944 (80.3% ) | 30015823 (86.0%) |
| WR_Rep3 | 41246195 | 41201748 | 36346256 (88.2%) | 34213672 (94.1%) |
| IS_Rep1 | 48068466 | 48044766 | 44113349 (91.8%) | 41579795 (94.3%) |
| IS_Rep2 | 59478846 | 59457013 | 53316450 (89.7%) | 50385449 (94.5%) |
| IS_Rep3 | 50461983 | 50431513 | 44309667 (87.9%) | 40862143 (92.2%) |
| IR_Rep1 | 49887025 | 49817323 | 34173228 (68.6%) | 27508298 (80.5%) |
| IR_Rep2 | 46524035 | 46388882 | 36484231 (78.6%) | 30791155 (84.4%) |
| IR_Rep3 | 44290885 | 44212427 | 33965812 (76.8%) | 26961392 (79.4%) |
| **96 hpi** |  |  |  |  |
| WS_Rep1 | 47917153 | 47859026 | 42620387 (89.1%) | 40114353 (94.1%) |
| WS_Rep2 | 39913474 | 39856580 | 36310353 (91.1%) | 34147293 (94.0%) |
| WS_Rep3 | 41393999 | 41356292 | 36976252 (89.4%) | 34756539 (94.0%) |
| WR_Rep1 | 38982060 | 38958960 | 34366848 (88.2%) | 31821355 (92.6%) |
| WR_Rep2 | 37540346 | 20819707 | 18823510 (90.4%) | 17206129 (91.4%) |
| WR_Rep3 | 46754139 | 46584494 | 41028501 (88.1%) | 37571920 (91.6%) |
| IS_Rep1 | 38736151 | 38701116 | 33794050 (87.3%) | 27419248 (81.1%) |
| IS_Rep2 | 46312609 | 46273257 | 40220581 (86.9%) | 35580415 (88.5%) |
| IS_Rep3 | 44795810 | 44747353 | 39415054 (88.1%) | 34854141 (88.6%) |
| IR_Rep1 | 42124052 | 42053185 | 36291748 (86.3%) | 33413595 (92.1%) |
| IR_Rep2 | 48423586 | 48360521 | 42502191 (87.9%) | 38207343 (89.9%) |
| IR_Rep3 | 48599374 | 48524812 | 39546746 (81.5%) | 34883610 (88.2%) |

^a^Rep=replication; WS=susceptible near isogenic line mock inoculated with water; IS= susceptible near isogenic line inoculated with *Colletotrichum lindemuthianum* race 73; WR= resistant near isogenic line mock inoculated with water; IR= resistant near isogenic line inoculated with *Colletotrichum lindemuthianum* race 73; hpi=hours post inoculation.
